# Biographical

**Published:** 1889-03

**Authors:** 


### Biographical.

Dr. Flavius Searle, a veteran of fifty years' practice of dentistry, finished his earthly career Sunday, the 10th, in the city of Springfield. Dr. Searle was largely known throughout the United States, and was much respected. Although he might be claimed as one of the old school practitioners, yet he was in more than an ordinary sense progressive. He was tenacious in his views and slow to accept new theories, being disposed critically towards all innovations in practice before he would adopt them. He was strongly conservative in his nature, and this, together with his other characteristics, made him an antagonist in many of the associations of his professional fellows. He was a man of gentlemanly demeanor and rather retiring, the latter trait often giving those who did not know him familiarly the impression of his being unsympathetic. But the doctor will be much missed in the local associations of New England. Having had a familiar acquaintance with him for some thirty-eight years, and knowing not a little of his restless voyage in life, we can say we believe that he has come to the placid sea of rest, and home at last. *Requiescat in pace.*

Dr. Searle was born in Southampton, Massachusetts, in 1814. His early education was directed in the interest of the ministry, but he was obliged to suspend his studies because of ill-health. Later he entered Amherst College, but physical frailties debarred him from the continuance of his studies. He then gave his attention to what was called the Thomsonian Practice of Medicine, but his mechanical attributes secured him for dental practice at last. After a short itinerancy which was common in that day, he opened an office at Springfield, in 1837. The doctor, with two others, Drs. Cyrus Perkins and Ames commenced practice in Springfield the same year. It may not be without profit to mention the fact, that but a few weeks since, Dr. Perkins, broken, blind and forsaken by his fellows, ended his career in the alms house at Springfield. We can only ask: Does not this progressive age suggest a larger fraternity? Dr. Searle was an honorary member of the Massachusetts Dental Society, and an active member of others. Dr. Searle leaves only one child, a daughter. By extreme frugality, the doctor accumulated something above $30,000. The funeral services were attended by thirty-five dentists, among them, were Drs. Post, Beales, Jones, Merliss, Noble, Derby, E. S. Hurlburt, J. S. Hurlburt, Stebbins, Bartholamew, L. D. Sheperd, McManus, Taylor, Parmalee, Andrews, Leitch, Davenport, Patten, Stockwell, Morgan, and Mills. The services were in charge of the Rev. Dr. Buckingham, assisted by Mr. Merriam, a personal friend, and by Dr. Searle's request, who read extracts from several authors, which were quite appropriate, and concluded with a well arranged eulogy, which will be well worth a perusal, and will doubtless be published. Some choice music was furnished by the Orpheus Glee Club.

A plain man and an enthusiastic admirer of Dr. Searle's (a dentist) remarked to us that "there was no other New England dentist left who would get any such send-off as the old doctor had." We think this does nearly complete the line of such types of men. Among these, we recall such names as Drs. S. P. Miller, Harwood, Tucker, Ball, Wilson, Hitchcock, Parmalee, Crofoot, Riggs, and a score more. All these largely represent the old school practice, some of them, more largely than others, have imbibed the progressive spirit—all these, have in a large degree honored their calling.

May all of us who remain, do as well, and much more. We are varied in our dispositions, going from extreme enthusiasm to the same in conservatism, both are absolutely essential, and in the order of divine control, they work together for good. What may be esteemed as fanaticism at the beginning of a century, becomes at the end pure conservatism. J. M., Feb. 15, '89.

